# Functional Outcomes of Robot-Assisted and Laparoscopic Partial Nephrectomy for cT1a and cT1b Renal Neoplasia with Low-Intermediate Tumor Complexity: Do Mass Localization, Ischemia, and Surgical Approach Matter?

**DOI:** 10.1245/s10434-025-18554-5

**Published:** 2025-10-28

**Authors:** Pier Paolo Prontera, Antonio Balestra, Marco Lattarulo, Gianluigi Califano, Francesco Di Bello, Claudia Collà Ruvolo, Simone Morra, Angelo Porreca, Luca Di Gianfrancesco, Arman Tsaturyan, Francesco Dibenedetto, Francesco S. Grossi

**Affiliations:** 1 Department of Urology, S.S. Annunziata Hospital, Taranto, Italy; 2 Department of Urology, Hospital Valle D’Itria, Martina Franca, Italy; 3 Department of Neurosciences, Reproductive and Odontostomatologic Sciences, University of Naples Federico II, Naples, Italy; 4 Department of Urology, Humanitas Gavazzeni Hospital, Bergamo, Italy; 5 Department of Urology, Yerevan State Medical University after Mkhitar, Heratsi, Yerevan, Armenia; 6 Department of Urology Erebouni Medical Center, Yerevan, Armenia; 7 Department of Operating Room, S.S. Annunziata Hospital, Taranto, Italy

**Keywords:** Renal neoplasm, Partial nephrectomy, Warm ischemia time, Renal function, Robotic surgery, Laparoscopic surgery, Nephrometry score

## Abstract

**Background:**

Nephron-sparing surgery is the gold standard for the treatment of cT1 renal neoplasms. The literature extensively debates the impact of various surgical techniques on long-term renal function, focusing particularly on ischemic damage and warm ischemia time (WIT). “Residual vascularized parenchymal volume” has emerged as a key factor influencing renal function outcomes. This study investigates the role of tumor localization and surgical technique (on-clamp versus off-clamp) in renal functional recovery for patients with low-to-intermediate tumor complexity underwent both robotic and laparoscopic partial nephrectomy.

**Patients and Methods:**

A multicenter retrospective study was conducted on 100 patients with cT1a or cT1b renal tumors (RENAL score 4–9) who underwent robotic or laparoscopic partial nephrectomy between 2020 and 2024. Demographic, tumor, and perioperative data were collected. Trend of serum creatinine and estimated glomerular filtration rate (eGFR) was evaluated at baseline, immediately postsurgery, and at 1, 3, 6, and 12 months. Statistical analysis was performed using Mann–Whitney U, chi-square, and Spearman’s rank correlation tests.

**Results:**

No significant differences in renal function were found between cT1a and cT1b tumors or between on-clamp and off-clamp techniques. The robotic and laparoscopic approaches also showed no significant difference in long-term renal outcomes.

**Conclusions:**

Tumor localization, ischemia type, and surgical approach did not significantly impact functional outcomes in low-to-intermediate complexity cT1 renal tumors. Comparable renal recovery was observed with both on-/off-clamp and robotic/laparoscopic techniques, as long as ischemia remained under 30 min. In this setting, the ischemic strategy may be chosen to enhance visibility and maximize parenchymal preservation.

Renal tumor enucleation has emerged as the gold standard for the surgical treatment of cT1 renal neoplasms. In recent decades, the literature has extensively debated the impact of various surgical techniques on long-term renal functional outcomes, particularly focusing on the role of ischemic damage and the impact of warm ischemia time (WIT) and tumor complexity on residual renal function.^1-5^ There is general consensus in the definition as “new baseline glomerular filtration rate” (NBGFR), described as the GFR measured 1–12 months after nephron-sparing surgery, thus excluding renal function fluctuations related to surgical stress and acute kidney injury (AKI).^6-8^ In the past few years, some studies evaluated the correlation between AKI and the risk of long-term renal impairment,^9-11^ although this occurrence is more frequent in patients with a solitary kidney.^11^ In this context, some authors explored the impact of the zero-ischemia technique on the residual renal function, with controversial results.^1,2,9-15^ However, recent studies have highlighted the concept of “residual vascularized parenchymal volume,” measured directly with advanced imaging techniques and dedicated software, as a key factor that may overshadow the impact of ischemia itself.^6,7,13-16^ According to this perspective, the role of ischemia in determining postoperative renal function seems to become significant only beyond certain ischemia thresholds, reported by some authors to be around 35 min.^17^ In this evolving scenario, it becomes essential to explore in which cases clampless surgery may be particularly advantageous. With the widespread use of high-resolution imaging, renal tumors are increasingly diagnosed at an early stage, often involving small tumor volumes (cT1a–cT1b).^18^ In these patients, the choice of surgical approach is often influenced by tumor complexity, typically summarized using nephrometry scores such as PADUA and RENAL. Deep or hilar lesions are generally considered to carry a higher risk of intraoperative bleeding, thus favoring on-clamp approaches.^3,17-20^ The increasing adoption of robot-assisted partial nephrectomy (RAPN), now considered the treatment of choice for nephron-sparing surgery, has dramatically improved surgical precision. The enhanced magnification of the surgical field and the superior dexterity of robotic instruments (compared with standard laparoscopy) enable precise, step-by-step dissection and selective coagulation of small vessels during enucleation, with better functional outcomes in robotic group as found by some authors.^21-24^ Within this context, the aim of our multicenter retrospective study was to address the following question: does tumor localization and surgical technique truly influence functional outcomes following nephron-sparing surgery in patients with low-to-intermediate tumor complexity?

## Patients and Methods

This multicenter retrospective study was conducted on a cohort of 100 patients diagnosed with cT1a or cT1b renal tumors with low-intermediate tumor complexity (RENAL score 4–6 and 7–9). All patients underwent robotic or laparoscopic partial nephrectomy between 2020 and 2024. No patients had a solitary kidney.

Demographic data, tumor characteristics (RENAL score and anatomical location), surgical technique (on-clamp or off-clamp, laparoscopic or robotic), and perioperative parameters (e.g., warm ischemia time, estimated blood loss) were collected from electronic medical records. Renal functional outcomes were evaluated using serum creatinine and estimated glomerular filtration rate (eGFR, CKD-EPI equation) preoperatively, immediately postoperatively, and at follow-up visits (1, 3, 6, and 12 months).

Comparisons of continuous variables were performed using the Mann–Whitney *U* test due to non-normal distribution. Categorical variables were analyzed using the chi-square test or Fisher’s exact test, depending on expected cell counts. Correlations between continuous variables were assessed with Spearman’s rank correlation coefficient. A two-sided *p*-value < 0.05 was considered statistically significant.

## Results

**Fig. 1:**
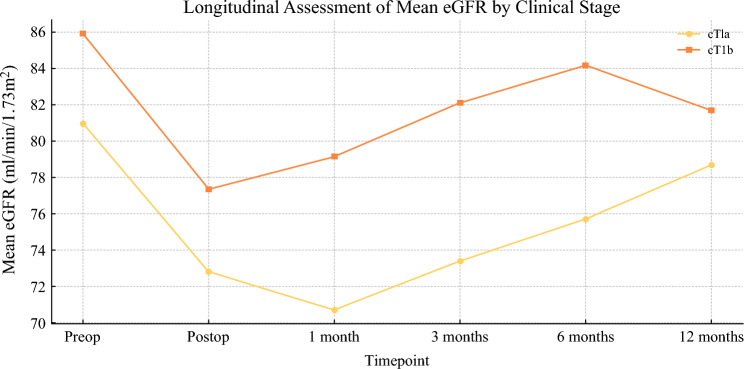
Longitudinal assessment of mean estimated glomerular filtration rate (eGFR) in patients with cT1a and cT1b renal tumors. Mean eGFR (ml/min/1.73 m^2^) is plotted for each time point (preoperative, postoperative, and follow-up at 1, 3, 6, and 12 months). No statistically significant differences were observed between cT1a and cT1b groups at any time point

**Fig. 2:**
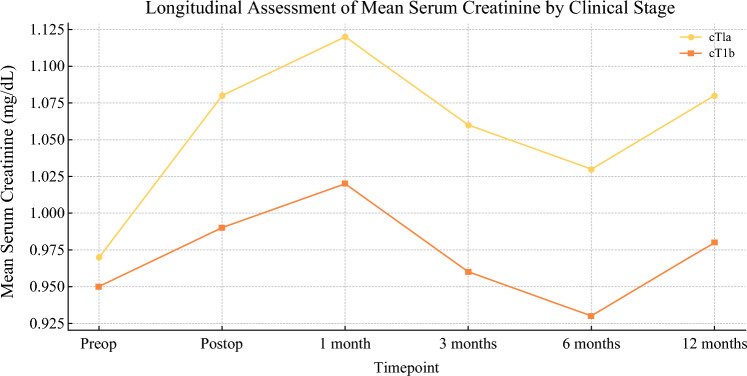
Longitudinal assessment of mean serum creatinine in patients with cT1a and cT1b renal tumors. Mean serum creatinine (mg/dL) is plotted for each time point. The data show similar trends in renal function preservation for both groups over the 12-month follow-up period

A total of 100 patients were analyzed, including 76 with cT1a tumors and 24 with cT1b tumors. The mean age was 59.9 ± 13.4 years for cT1a and 58.1 ± 11.7 years for cT1b (*p* = 0.417). Body mass index (BMI) did not differ significantly between groups (26.7 ± 3.1 kg/m2 for cT1a versus 26.2 ± 2.9 kg/m2 for cT1b, *p* = 0.843). However, the Charlson Comorbidity Index (CCI) was significantly higher in patients with cT1a tumors (5.1 ± 1.6 versus 3.7 ± 1.2, *p* = 0.007). Prevalence of hypertension and diabetes did not show significant differences (hypertension: 45% versus 42%, *p* = 0.721; diabetes: 16% versus 13%, *p* = 0.733). Mean RENAL score was significantly higher in cT1b tumors (6.5 ± 1.3 versus 5.9 ± 1.2, p = 0.003). Most cT1a tumors were in the low-complexity RENAL class (4–6: 76.3%) compared with cT1b tumors (58.3%). Conversely, the moderate-complexity RENAL class (7–9) was more prevalent in cT1b tumors (41.7% versus 23.7%). Tumor location analysis revealed that hilar tumors were more frequent in cT1b (58.3%) than cT1a tumors (47.4%, *p* = 0.013). Polar inferior tumors were exclusively seen in patients with cT1a tumors (28.9% versus 0%, *p* = 0.001). No significant differences were found for polar superior (*p* = 0.089), mediorenal (*p* = 0.682), anterior (*p* = 0.243), or posterior (*p* = 0.233) locations. Warm ischemia time (WIT) and estimated blood loss were comparable between cT1a and cT1b tumors (WIT: 16.7 ± 5.3 versus 15.9 ± 4.9 minutes, *p* = 0.325; blood loss: 365.5 ± 210.4 versus 377.5 ± 220.6 ml, *p* = 0.647). No significant differences were observed for the use of renorrhaphy (72.4% versus 79.2%, *p* = 0.503) or the presence of positive surgical margins (2.6% versus 4.2%, *p* = 0.659) [Table 1]. Across all time points up to 12 months, no statistically significant differences were found in eGFR or serum creatinine between the cT1a and cT1b groups. While eGFR tended to be slightly higher and creatinine levels slightly lower in the cT1b group, these differences did not reach statistical significance (all *p*-values > 0.05) [Table 2]. Median eGFR at 12 months was 83.2 ml/min/1.73m2 for laparoscopic surgery and 75.3 ml/min/1.73m2 for robotic surgery (*p* = 0.202) [Fig. 1]. Median serum creatinine at 12 months was 0.95 mg/dL for laparoscopic surgery and 1.0 mg/dL for robotic surgery (*p* = 0.383) [Fig. 2] [Table 3]. Furthermore, analysis of the distribution of acute kidney injury (AKI) within RENAL nephrometry score classes showed no significant differences between cT1a and cT1b tumors across the low (4–6) and intermediate (7–9) complexity groups. Specifically, 14 AKI cases were observed in the cT1a tumor group and two in the cT1b tumors group within the 4–6 complexity class (*p* = 0.494). In the 7–9 class, five AKI cases were observed in cT1a tumors, while none were found in cT1b tumors (*p* = 0.533). Analysis of tumor localization revealed that polar inferior tumors were significantly more frequent in cT1a tumors compared with cT1b tumors (28.9% versus 0%, *p* = 0.001), while hilar tumors were significantly more frequent in cT1b tumors (58.3% versus 47.4%, *p* = 0.013). No significant differences were observed in the polar superior, mediorenal, anterior, or posterior locations [Table 4]. Finally, subgroup analysis within the low-complexity RENAL class (4–6) showed significant differences in the distribution of cT1a and cT1b tumors depending on the ischemia technique used. Specifically, in the off-clamp group, 86% of cT1a and 100% of cT1b tumors were included (*p* = 0.000), while in the on-clamp group, 60% of cT1a and 14.3% of cT1b tumors were included (*p* = 0.000). For moderate complexity tumors (RENAL 7–9), no significant differences in distribution were found between cT1a and cT1b tumors across either ischemia technique [Table 5].

**Table 1:** Comparative analysis of demographic, clinical, tumor location, and perioperative variables between patients with cT1a and cT1b renal tumors

Variable	cT1a (*n* = 76)	cT1b (*n* = 24)	*p*-value
Age (years), mean (SD)	59.9 (± 13.4)	58.1 (± 11.7)	0.417
BMI (kg/m^2^), mean (SD)	26.7 (± 3.1)	26.2 (± 2.9)	0.843
Charlson Comorbidity Index, mean (SD)	5.1 (± 1.6)	3.7 (± 1.2)	0.007
RENAL Score, mean (SD)	5.9 (± 1.2)	6.5 (± 1.3)	0.003
RENAL 4–6, *n* (%)	58 (76.3%)	14 (58.3%)	0.147
RENAL 7–9, *n* (%)	18 (23.7%)	10 (41.7%)	0.147
RENAL 10–12, *n* (%)	0 (0%)	0 (0%)	–
Polar score			
Polar superior, *n*(%)	40(52.6)	18(75)	0.089
Pola Inferior, *n*(%)	22(28.9)	0(0)	0.001
Mediorenal, *n*(%)	14(18.4)	6(25)	0.682
Tumor location			
Anterior, *n*(%)	2(2.6)	2(8.3)	0.243
Posterior, *n*(%)	38(50)	8(33.3)	0.233
Hilar, *n*(%)	36(47.4)	14(58.3)	0.013
Warm ischemia time (minutes), mean (SD)	16.7 (± 5.3)	15.9 (± 4.9)	0.325
Estimated blood loss (ml), mean (SD)	365.5 (± 210.4)	377.5 (± 220.6)	0.647
Preoperative eGFR (ml/min/1.73m2), mean (SD)	84.2 (± 19.5)	80.1 (± 18.7)	0.351
Preoperative creatinine (mg/dL), mean (SD)	0.95 (± 0.21)	0.98 (± 0.19)	0.522
Male sex, *n* (%)	52 (68.4%)	18 (75.0%)	0.541
No Renorrhaphy, *n* (%)	55 (72.4%)	19 (79.2%)	0.503
Surgical complications, *n* (%)	11 (14.5%)	4 (16.7%)	0.783
Positive surgical margins, *n* (%)	2 (2.6%)	1 (4.2%)	0.659

Data are presented as mean (standard deviation) for continuous variables and as number (percentage) for categorical variables. Subgroups of the RENAL score (4–6, 7–9, 10–12) are also reported descriptively without *p*-values due to limited sample sizes. Continuous variables were compared using the Mann–Whitney *U* test given their non-normal distribution (verified with the Shapiro–Wilk test). Categorical variables were compared using the chi-square test or Fisher’s exact test when appropriate (expected frequencies < 5). A two-sided *p*-value < 0.05 was considered statistically significant

**Table 2:** Longitudinal assessment of renal function (eGFR and creatinine) in patients with cT1a and cT1b renal tumors

Time point	eGFR cT1a (mean/median/range)	eGFR cT1b (mean/median/range)	eGFR *p*-value	Creatinine cT1a (mean/median/range)	Creatinine cT1b (mean/median/range)	Creatinine *p*-value
Preoperative	80.97/81.6/28.0–136.0	85.92/88.3/29.4–110.5	0.240	0.97/0.9/0.6–2.3	0.95/0.86/0.65–2.09	0.485
Postoperative	72.81/70.9/28.4–139.0	77.36/78.3/55.3–104.0	0.228	1.08/1.01/0.57–2.15	0.99/0.98/0.8–1.31	0.445
1 month	70.72/74.55/18.1–125.3	79.15/80.9/52.7–101.7	0.126	1.12/1.03/0.66–2.49	1.02/1.02/0.78–1.32	0.625
3 months	73.41/77.35/19.5–125.5	82.11/84.9/56.3–106.5	0.139	1.06/0.96/0.61–2.46	0.96/0.95/0.71–1.27	0.539
6 months	75.72/78.95/20.6–130.0	84.18/87.15/58.6–107.8	0.160	1.03/0.94/0.6–2.42	0.93/0.94/0.68–1.25	0.545
12 months	78.7/75.8/28.6–159.3	81.69/80.15/55.5–110.2	0.429	1.08/1.0/0.6–2.23	0.98/0.96/0.72–1.28	0.485

Data are expressed as mean/median/range (minimum–maximum). eGFR is reported in ml/min/1.73 m^2^, and creatinine in mg/dL. *p*-values were calculated using the Mann–Whitney *U* test to compare renal function metrics between the two clinical stage groups (cT1a versus cT1b) at each time point

**Table 3:** Comparison of renal functional recovery at 12 months between laparoscopic (A) and robotic (B) surgery

Parameter	Laparoscopy (A)	Robotic surgery (B)	*p*-value
eGFR at 12 months (ml/min/1.73m2)	Median: 83.2	Median: 75.3	0.202
Serum creatinine at 12 months (mg/dl)	Median: 0.95	Median: 1.0	0.383

Data are presented as medians. No statistically significant differences were observed for eGFR or serum creatinine at 12 months between the two surgical approaches (*p*-values calculated using Mann–Whitney *U* test)

**Table 4:** Comparison of RENAL nephrometry score classes (4–6, 7–9, 10–12) between cT1a and cT1b tumors, stratified by anatomical tumor location

Location	RENAL class	cT1a (n)	cT1b (n)	*p*-value
Polar superior	4–6	34	12	0.161
	7–9	6	6	0.161
	10–12	0	0	–
Polar inferior	4–6	14	0	1.000
	7–9	8	0	1.000
Mediorenal	4–6	10	2	1.000
	7–9	4	4	0.235
	10–12	0	0	–
Anterior	4–6	2	2	1.000
	7–9	0	0	–
Posterior	4–6	26	4	0.174
	7–9	12	4	1.000
	10–12	0	0	–
Hilar	4–6	22	6	0.309
	7–9	8	8	0.309

Data are presented as the number of tumors in each RENAL score class for both clinical stages (cT1a and cT1b). *p*-values were calculated for each comparison using Fisher’s exact test. N/A indicates cases where no *p*-value could be computed due to absence of tumors in one or both groups. This detailed analysis allows for a comprehensive understanding of how tumor complexity (as measured by the RENAL nephrometry score) varies between cT1a and cT1b tumors across different anatomical locations.

**Table 5:** Comparison of RENAL nephrometry score classes (4–6, 7–9, 10–12) between cT1a and cT1b tumors, stratified by ischemia technique (on-clamp versus off-clamp)

RENAL class	Ischemia type	cT1a (*n*, %)	cT1b (*n*, %)	*p*-value
4–6	Off-clamp	43 (86%)	13 (100%)	0.000
4–6	On-clamp	15 (60%)	1 (14.3%)	0.000
7–9	Off-clamp	8 (14%)	4 (30.8%)	0.22
7–9	On-clamp	10 (40%)	6 (85.7%)	0.289

Data are expressed as absolute numbers and percentages within each subgroup. *p*-values were calculated using Fisher’s exact test for each comparison.

## Discussion

The aim of our study was to address the following question: does tumor localization and surgical technique truly influence functional outcomes following nephron-sparing surgery in patients with low-to-intermediate tumor complexity? Despite differences in baseline tumor characteristics, such as a higher RENAL score in cT1b tumors and the predominance of hilar location in cT1b cases, our findings show no statistically significant differences in renal functional recovery at 12 months postsurgery between the cT1a and cT1b groups. In particular, although off-clamp surgery was more frequently performed in cT1b tumors within the low-complexity RENAL class and hilar tumors were more common in cT1b cases, these factors did not translate into significant differences in postoperative eGFR or serum creatinine levels. These results align with emerging evidence suggesting that while warm ischemia time (WIT) and ischemia remain important considerations, their significance becomes more pronounced only beyond certain thresholds (e.g., > 35 min),^17^emphasizing the concept of “residual vascularized parenchymal volume” as a primary factor in residual renal function following nephron-sparing surgery.^6,7,16-19,25-29^ In real-world practice, especially for low-to-moderate complexity tumors, surgical precision, meticulous resection, and parenchymal preservation may play a more decisive role in long-term renal function than the ischemic technique itself. Notably, our data suggest that even in small renal masses (cT1a), the use of an on-clamp technique may be justified and potentially advantageous in terms of maximizing visualization and enabling precise resection to preserve as much healthy parenchyma as possible. This approach could help mitigate the risk of inadvertent damage during tumor excision, thereby preserving renal function. Moreover, when comparing laparoscopic and robotic approaches, no statistically significant differences in long-term functional outcomes were observed in our cohort. This contrasts with other studies, including our prior work,^24^ which reported a clear functional advantage for robotic surgery, particularly in more complex tumors. The lack of difference in our cohort may reflect the relatively low-to-intermediate complexity of the tumors studied, where the robotic platform’s advantages, such as enhanced precision and improved visualization, may be less critical in achieving excellent functional outcomes.^22,23^ However, this hypothesis warrants further evaluation in larger studies with more robust sample sizes to confirm or refute these observations. Based on the observations obtained during this study, it appears that for functional outcomes in patients with renal tumors classified as T1 with a low-to-intermediate RENAL risk, the choice between an on-clamp or off-clamp technique, whether robotic or laparoscopic, is largely indifferent. However, it is important to emphasize that all patients undergoing ischemia had WIT times of less than 30 min, and laparoscopic procedures were performed by highly experienced surgeons in this type of minimally invasive surgery. Our findings are consistent with the evolving paradigm highlighted by Pandolfo et al., who emphasized the importance of careful decision-making in complex renal masses where partial nephrectomy may provide both oncologic and functional advantages over radical nephrectomy.^30^ In conclusion, based on the observations reported, we believe it is reasonable to conclude that in patients with renal tumors classified as T1 with low-to-intermediate RENAL risk, the on-clamp surgical technique appears justified to improve field visibility and clarity, thereby minimizing damage to healthy renal parenchyma, both with robotic and laparoscopic techniques (if performed by highly experienced laparoscopic surgeons), provided that ischemia times remain under 30 min. It is important to acknowledge the retrospective nature of our study and the limited sample size, which may limit the generalizability of these findings. Larger, prospective studies are needed to confirm these results and further elucidate the interplay between ischemic technique, tumor complexity, surgical approach, and long-term renal functional recovery.

## Conclusions

Tumor localization and surgical technique do not appear to have a significant impact on functional outcomes following nephron-sparing surgery in this patient group. Both on-clamp and off-clamp techniques, as well as robotic and laparoscopic approaches, can result in similar renal functional recovery outcomes, provided ischemic times are kept short (< 30 min). In this context, warm ischemia could be considered for any T1 renal tumor with a low-to-intermediate RENAL risk undergoing nephron-sparing surgery, as it may enhance dissection precision and minimize damage to healthy parenchyma, ultimately preserving the new baseline glomerular filtration rate (NBGFR) and renal function.

## Data Availability

The data associated with the paper are not publicly available but are available from the corresponding author on reasonable request.
